# Archaeometallurgical characterisation of ancient copper slags from pre‐Harappan site, Kunal, India

**DOI:** 10.1002/ansa.202100050

**Published:** 2022-07-20

**Authors:** Aditya Prakash Kanth, Manager Rajdeo Singh, Buddha Rashmi Mani

**Affiliations:** ^1^ Centre for Heritage Management Ahmedabad University Ahmedabad India; ^2^ National Research Laboratory for Conservation of Cultural Property Lucknow India; ^3^ National Museum Institute of History of Art Conservation and Museology New Delhi India

**Keywords:** copper, Harappan, ICP‐MS, Kunal, SEM‐EDX, Slag, XRD, XRF

## Abstract

The copper slags collected during the excavation of the Early Harappan period site at Kunal in northern India were studied to understand the advancement of smelting technology and the achieved smelting temperature in the furnace by undertaking archaeometallurgical characterisation of the slags. In this research, two types of slags such as slag with glassy appearance and granulated slag were selected for the study. The microscopic structure and distribution of slag components were investigated using optical microscopy and phase determination was done by X‐ray diffraction (XRD). Chemical characterization of the slags was conducted using scanning electron microscopy–energy‐dispersive X‐ray spectroscopy, X‐ray fluorescence analysis (XRF) and inductively coupled plasma–mass spectrometry to build a complete chemical profile of the slags. Fayalite and magnetite were the dominant phases in the glassy slag; however, the granulated slag showed the dominance of calcite as secondary phase which reflected the dolomitic stoichiometry of the slag. The presence of dominant fayalite mineral phase as detected by XRD and higher concentration of iron as detected by XRF indicated the reducing environment during the smelting process. In this study, the absence of sulphur is reported which is unique to the ancient copper slag.

## 1 INTRODUCTION

Copper was widely used in India from the 3rd to 4th century BC, as mentioned in some of the accounts of Greek ambassador Megasthenes’ India visit in 302 BC.^1^ Many recovered copper and bronze sculptures from similar excavation sites can be found in museums, demonstrating the common practice of extracting copper from its ores and using it to make different implements. Slag, a waste product resulting from the pyrometallurgical extraction of the metal,^2^ is one of the most important resources for understanding metal extraction technology, type of furnace, smelting environmental condition and the efficiency of the smelting process to reduce pure metals from their ores contained on those archaeological sites. The most ancient slags that have been found anywhere are chalcolithic copper slags. The limited capacity furnace discovered in the Aravalli site in northern India showed that the furnace was built on bricks with brick side walls and mud on both sides, which helped in reaching and retaining intense heat in the furnace needed for smelting.^3^ Similar smaller kilns with vitrified inner mud surfaces and high‐quality copper ingots were discovered at the Lothal site in Gujarat, India, during the same era of the Indus Valley Civilisation, indicating the sophistication of copper smelting technology in the region.^3^ Due to the open furnace's inability to hit the necessary temperature (1083°C) for copper melting, evidence has been discovered that the furnaces were drilled slightly deeper to achieve the required temperature and allow oxidized metal ores to be melted.^3^

Copper occurs generally in association with oxygen, sulphur, carbonates and/or other transition metals.^4^ Copper slags are usually composed of CuO_2_, CuO, Cu_2_S, etc., and also consist of some other metals such as nickel, iron, cobalt, etc., which are also used as a secondary source of metal extraction and not as waste material.^5^, ^6^ Elemental fingerprinting is a useful technique for understanding the history of copper production or ore smelting technology since it aids in determining the source of the ores and future trade routes.^7^ Generally, the determination of the chemical composition of slags is done using X‐ray fluorescence analysis (XRF) and scanning electron microscopy–energy‐dispersive X‐ray spectroscopy (SEM‐EDX) whereas X‐ray diffraction (XRD) is very useful for phase determination. For estimation of trace elements in ancient metals in ppm and ppb, inductively coupled plasma–mass spectrometry (ICP‐MS) and laser ablation‐ICP‐MS have been very useful.^8^ It aids in the identification of isotopes and elemental analysis. ICP‐MS has proven to be a very useful tool for determining trace elements because the detection limits are extremely low, ranging from parts per million to parts per billion. Even though it is an invasive method of study, it only involves micro‐sized samples and minimal sample preparation.^9^

## 2 MATERIALS AND METHODS

**FIGURE 1:**
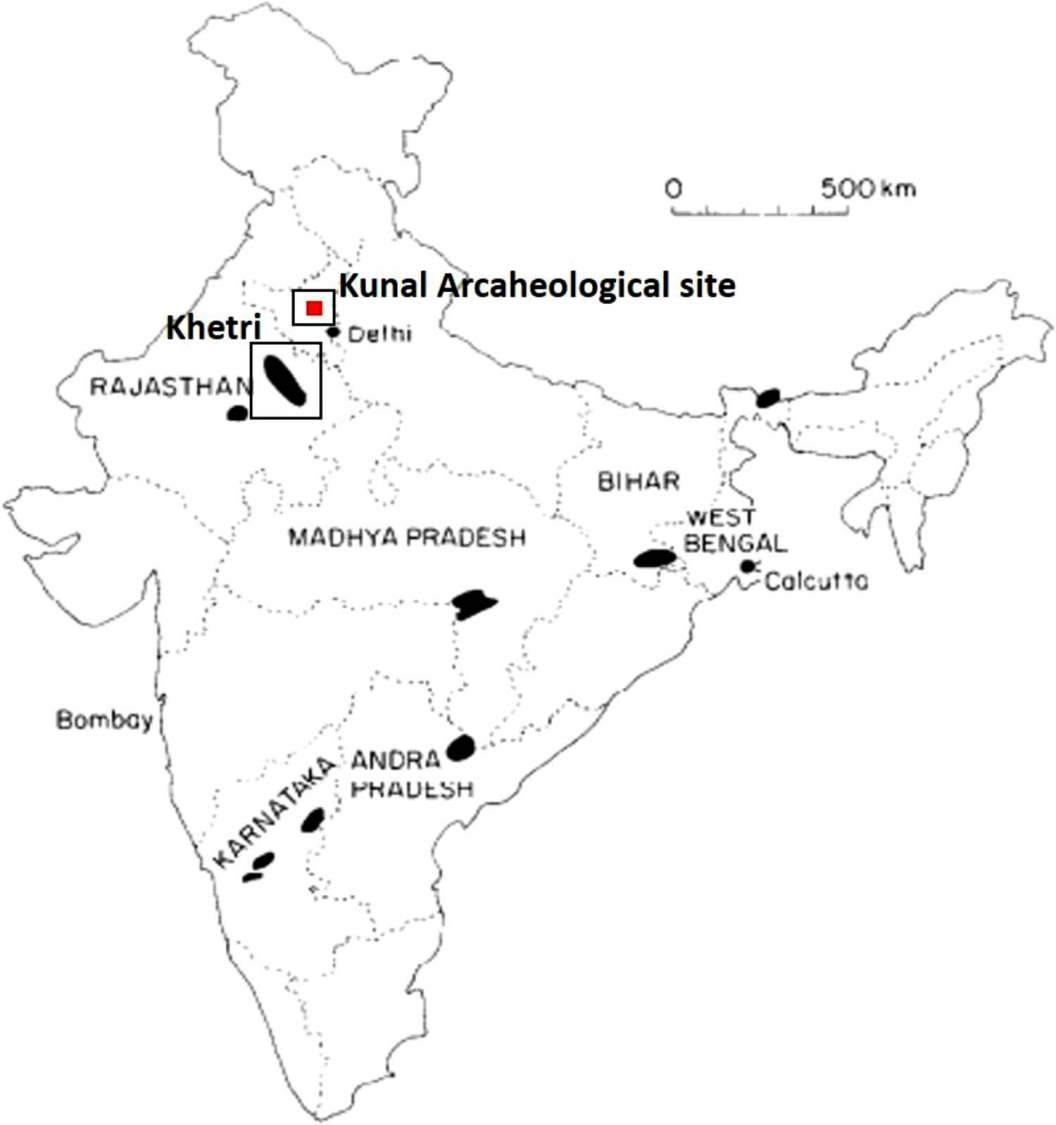
Map of India showing Kunal archaeological site where the slag was obtained and the nearby ancient copper mines at Khetri

Kunal is considered a chalcolithic site situated in the Fatehabad district of the northern state of Haryana, India (Figure 1). Various kinds of objects and material pieces were collected from several excavations which proved highly informative about the material and the cultural traditions of the early period of the Indus Valley Civilization.^10^, ^11^ Just a few slags were discovered at the site, indicating that smelting was not a continuous but rather sporadic operation. It also suggests that copper implement output was lower in this region^12^, ^13^ since the Khetri mines were far away from the smelting location. However, there is every possibility that the ores for copper smelting were brought from the Khetri copper belt which is stretched for over 100 km in the Aravalli mountains and is the largest copper ore deposit in India.^4^

**FIGURE 2:**
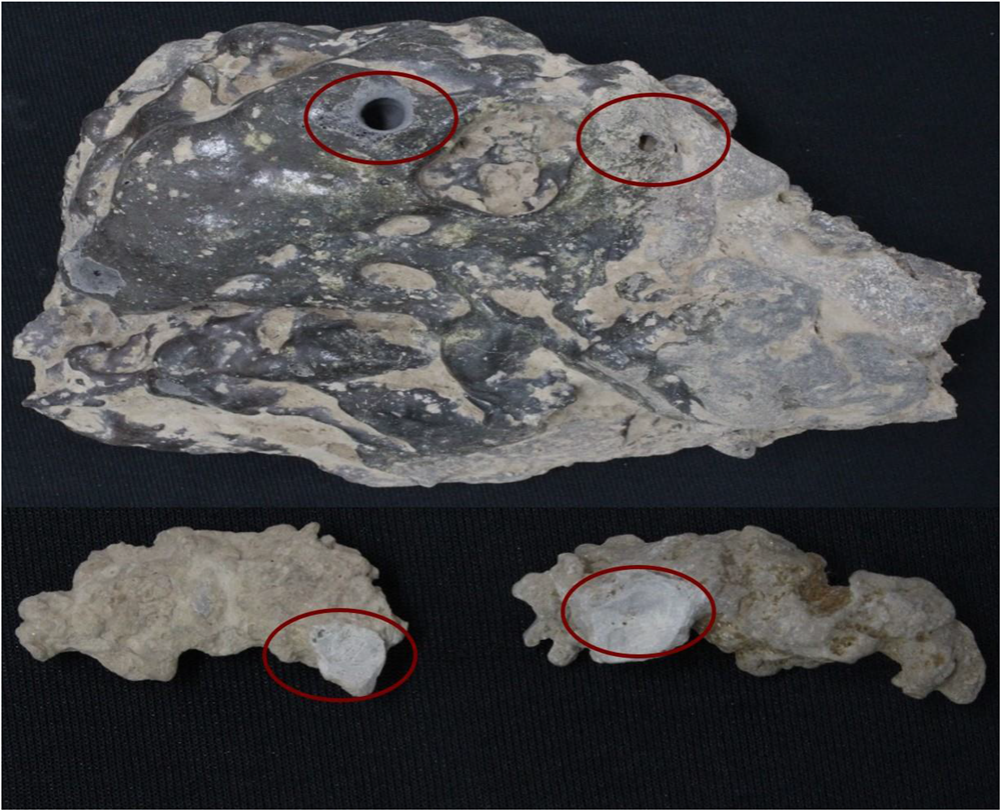
Primary glassy slag on top and two secondary slags on the bottom. Circled are the places from where samples were taken

The study aimed to classify the collected slags, learn more about the smelting process and map out the entire chemical profile, their mineral distribution, phase morphology, granulometry, etc. Metal slag samples in this study were discovered in the trenches alongside the potsherds during the recent excavation. A solid metal mass was discovered, and some smaller bits of high frothed slag were collected. The slags were cleaned with distilled water using a soft brush to dislodge the loosely adherent dust and dirt. Two types of slags were investigated in this study. The slags were micro‐drilled at four different points and powdered samples were obtained (Figure 2).

### 2.1 Instrumentation

SEM‐EDX analysis, Carl Zeiss EVO 50 scanning electron microscope was used at various magnifications at high vacuum mode (coupled with a Bruker EDX instrument) for the study of elemental composition and microstructure. The accelerating voltage was set at 20 kV at a working distance of 8 mm at a resolution of 2250 nm and the EDX data was processed with Roentag software. All the samples were uncoated.

For ICP‐MS analysis, 300 mg of four powder samples were taken and dissolved in aqua regia v/v and the final volume of the sample was 40 ml. The digested samples were analyzed using Agilent 7900 ICP‐MS where the samples were ionized and the ionized mass was directed to the MS detector. Data was collected with Agilent ICP‐MS Mass Hunter software that had several plasma modes present which included the Ultra High Matrix Introduction dilution factor. Data were acquired in count per second.

Micro‐XRF analysis of the samples was carried out by micro‐XRF (model ARTAX 200; Bruker, Germany) at a voltage of 50 kV at 700 mA current, and the data collection time was 300 live seconds. The results are reported in the form of elements. The voltage was further reduced to 20 kV to get an accurate measurement of metals like Cu, Fe, Mn, etc.^14^

For the mineralogical composition, X‐ray diffraction analysis was conducted on a BRUKER AXS SMART APEX diffractometer with a charge‐coupled device area detector KR, 0.71073 Å. The powdered samples collected by drilling the slag were further ground with mortar and pestle. Collection time was 30 min per scan. X‐ray diffractogram was recorded in the 2*θ* range of 10–80° at room temperature 40 kV current was applied.

## 3 RESULTS AND DISCUSSION

### 3.1 Appearance

The appearance of the large lump of the slag was very fine‐grained, homogenous and glassy appearance. The slag's black colour and glassy appearance meant that it had been slowly cooled in the open air and had higher specific gravity. This meant that this slag lump was the first layer of slag (primary slag) produced during the smelting process.^5^, ^15^, ^16^ During the micro‐drilling sampling process, some voids in the slag were discovered that were not visible on the slag's external surface. Until flotation, the smelter slag had to be cooled slowly, naturally. The two other smaller slags were grey and appeared granulated and amorphous, which may have resulted from the slag's rapid quenching. These slags were considered the second layer of slag in this study; these slags were classified as secondary slags.

### 3.2 Optical microscopic examination

**FIGURE 3:**
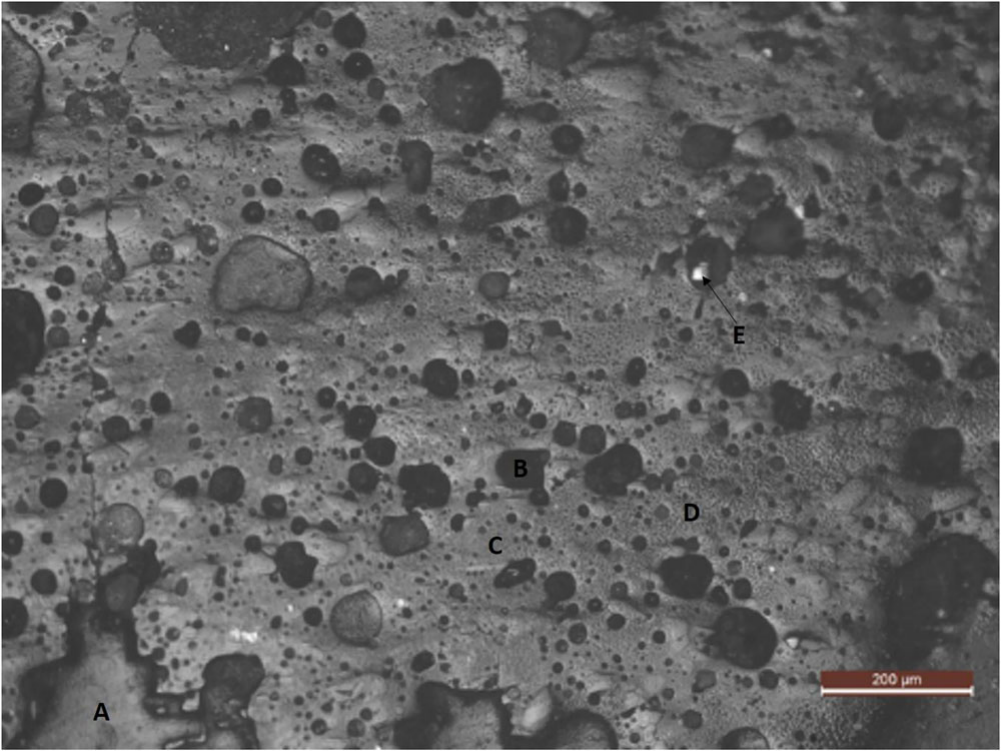
Optical microscopic image of the glassy slag showing (A) iron oxides, (B) dark grey fayalite, (C) light grey glassy matrix, (D) Numerous cavities and micro‐bubble generation due to sulphur dioxide and (E) copper micro‐prills

Figure 3 shows the plenty of shiny silvery islands of iron oxide present in the light grey glassy matrix. The dark spots of fayalite are also prominent throughout the matrix. The microscopic copper prills can also be seen in tiny white spots in a few areas. In this glassy matrix, gas cavities and microbubbles are also seen which seem to be in the range of 10–50 μm which could be sulphur dioxide microbubbles that were produced during the oxidization in the matte phase. These microbubbles first appear at the immiscible fluids' interface and gradually coalesce, giving rise to larger bubbles that spread across the slagging process. These rising microbubbles caused matte entrainment in the slag by moving matte droplets onto the slag's surface. The matte entrainment due to the rising gas bubbles depends on two things: first the larger bubble buoyancy force than the matte droplet drag force, and then the thickness of the matte film. It is the bubble buoyancy force that lifts the matte droplets.^17^ The above statement is presented in the equation below:

(1) $$\frac{d_{g}}{d_{matte}}=\sqrt[{3}]{\frac{V_{g}}{V_{matte}}}>\sqrt[{3}]{\frac{\rho_{matte\;}\_\;\rho_{slag}}{\rho_{slag}}}$$

($V_{g}$ and $V_{matte}$ = the bubble and matte volumes; $d_{g}$ and $d_{matte}$ = the gas bubble and matte droplet diameters; $\rho_{matte\;}$ and $\rho_{slag}$ = matte and slag densities).

Since both bottom‐blown and side‐blown techniques were used in ancient Indian smelting techniques, likely, the matte was mechanically entrained from the smelting furnace. The reaction zone and the settlement zone are the two main areas in a smelting furnace. The matte in the reaction zone goes through rapid oxidization, reaches the settlement zone and gets discharged.^17^ The above‐mentioned process of sulphur dioxide micro‐bubble generation in settlement zone during matte phase is presented below:

(2) $$\begin{array}{ccc} & & {\mathrm{CuFeS}}_{2}\left(s\right)+O_{2}\left(g\right)+{\mathrm{SiO}}_{2}\left(s\right)\to \left(\mathrm{Cu}-\mathrm{Fe}-S\right)\left(l\right)+\mathrm{FeO}-{\mathrm{SiO}}_{2}\left(l\right)\\ & & \;+\;{\mathrm{SO}}_{2}\left(g\right)+\mathrm{heat} \end{array}$$

### 3.3 Phase composition

**FIGURE 4:**
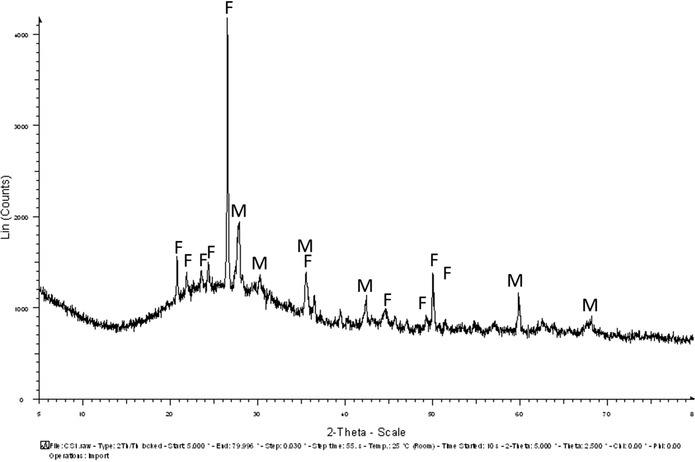
X‐ray diffraction (XRD) diffraction of the primary slag (glassy slag) showing two prominent phases, fayalite (F) and magnetite (M)

**FIGURE 5:**
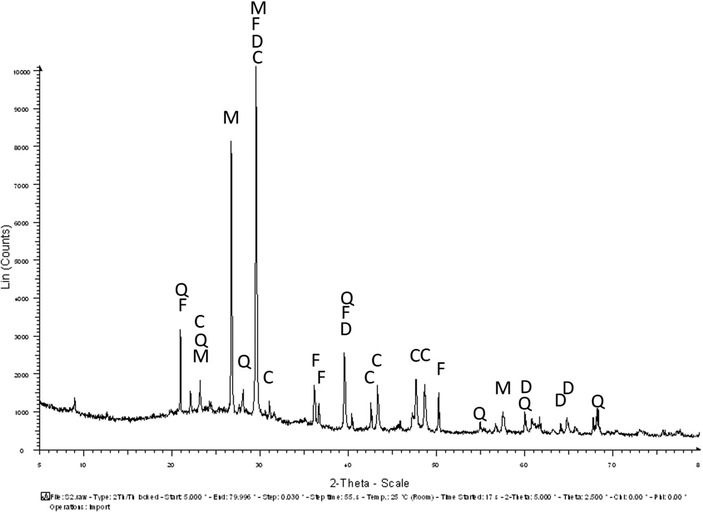
X‐ray diffraction (XRD) diffraction of the secondary slag (granulated slag) with identified phases as Q: Quartz; F: Fayalite; C: Calcite; D: Diopside; M: Magnetite

During the cooling process of slag, crystallization of the molten slag results in the formation of certain phases depending on the conditions of cooling and the addition of fluxes that are based on silicon, magnesium, calcium and aluminium.^18^ In the XRD diffractogram of the glassy slag (Figure 4), the prominent phases detected were fayalite (2FeO.SiO2) and magnetite (Fe3O4).^19^ The presence of magnetite could be due to the oxidation of iron oxide.^5^, ^20^ The presence of magnetite could also be partially due to the partial decomposition of fayalite into magnetite. In the granulated slag sample (Figure 5), the secondary calcite phase is very dominant. Apart from calcite, the other phases present were fayalite, magnetite, diopside and quartzite. The fayalite phase is more prevalent in the glassy slag sample than in the granulated slag sample. According to the XRD results, the dominant crystalline mineral is fayalite, and the dominant phase is calcite. The presence of fayalite and magnetite is due to the iron oxide being over‐oxidized.^5^ The presence of fayalite also indicates the smelting process taking place in reducing conditions. In the granulated slag, the silica and calcite content is quite high which suggests the amorphous phase of the slag. The high content of silica also indicates the fast cooling process of the slag.^5^, ^20^

Slags rarely corrode since the metals in them are still in their oxide state, which is the most stable form.^2^ When silica is applied to oxides during smelting, it forms silicate anions by reacting with oxides, which are tightly bonded in the slagging process.^2^ It was not very clear whether the silica was intentionally applied to the matte to isolate copper or whether it was already there when the ore concentrate was exposed to the reaction zone. However, it seems that silica was additionally added as a fluxing material to facilitate better smelting. Surprisingly, no sulphur was detected in any of the samples after XRF and EDX examination, and it was also not visible in optical microscopic images. If sulfide had been present, it would have emerged as a covalent matte phase separate from the silicate phase.^2^ Sulphur might have escaped completely from the gas cavities after the microbubble cavity formation due to the evolution of the sulphur dioxide gas.

### 3.4 Chemical composition

For investigating the chemical composition, XRF, SEM‐EDX and ICP‐MS analyses were carried out to get a complete chemical profile of the slags. For the XRF analysis, the samples were taken from the upper surface of the slags, the inner surface of the slags (after cutting and removing the upper surface) and after grinding the sample. Table 1 summarises the mass percentages of the elements in samples of the slags. It confirmed the presence of iron, copper, titanium, calcium, potassium, lead and silicon in both types of slags. The bulk analysis shows an exceedingly high level of iron in both types of slags. In the glassy slag sample, calcium, strontium and rubidium were found in high concentrations whereas, in granulated slag, rubidium and strontium were absent. In the granulated slag, XRF did not show any presence of manganese, whereas it was present in a smaller quantity in the glassy slag. Since these slags also showed some valuable metals such as titanium, these are also considered secondary sources of metals.^5^ The result of the XRF showed a very high concentration of iron which was due to the piling of Fe sum peaks that interfered with Pb as a result of the matrix effect. Therefore overlapping corrections were done to get the correct concentration of Fe and Pb. However, spectral overlapping of K‐lines and L‐lines could not be completely resolved as the distinguishing between these two lines were complicated due to their occurrence in low energy region. For the determination of the elemental composition, EDX was done and the major and minor elements detected from SEM‐EDX have been presented in Table 2. The SEM‐EDX spectra detected high percentages of oxygen, silicon, lead and aluminium. The very high weight percentages of oxygen indicate that all the elements detected are in oxide forms and the principal oxide was SiO_2_. Based on all the elements detected, EDX indicated a strong presence of the fayalite phase (Fe_2_SiO_4_). The presence of aluminium, magnesium, zinc and calcium can also be considered isomorphic impurities in the chemical composition of fayalite.^18^ Interestingly, sulphur was not detected in the EDX graph but the ore was rich in Pb. This probably also indicates the sophistication of the smelting process where all entrapped sulfides could be removed from the ore and the metal. The sulfides formed the micro‐bubbles in the matte which rose to the surface and escaped forming the micro‐cavities in the slag. This probably also indicates that the ancient people were highly skilled in refining copper metal where they could reduce the copper ores to a great extent. In early scientific investigations, sulphur was found in most of the metallurgical investigations.^21^, ^22^ XRF and EDX showed some variations in the result like XRF showed a very high concentration of iron in all samples whereas EDX showed iron only in smaller percentages. Likewise, Pb was found in high concentration in EDX whereas XRF showed it in smaller quantity. This could be due to the heterogeneity of the excavated slag samples.

**TABLE 1:** Mass percentages of elements obtained with X‐ray fluorescence analysis (XRF) analysis

**Samples**	**Si**	**K**	**Ca**	**Ti**	**Cr**	**Mn**	**Fe**	**Ni**	**Cu**	**Zn**	**Rb**	**Sr**	**Pb**	**As**
Granulated slag‐ upper surface	1.03	3.54	22.5	3.30	0.93	–	56.76	0.65	2.60	1.40	–	–	6.30	0.80
Granulated slag‐ after the cut	0.67	2.30	24.56	2.76	0.86	–	52.88	0.60	1.57	1.92	–	–	7.85	0.89
Granulated slag ‐ after the grind	0.75	3.22	22.35	2.50	0.99	–	55.61	0.60	1.34	1.14	–	–	7.57	0.96
Glassy slag ‐ upper surface	1.60	2.75	5.10	2.19	–	2.31	68.13	–	5.51	1.62	1.81	1.41	8.12	–
Glassy slag ‐ after the cut	0.89	2.33	7.63	2.46	–	2.38	63.74	–	6.86	1.41	2.07	1.28	8.84	0.93
Glassy slag ‐ after the grind	2.05	2.86	5.17	1.97	–	2.06	67.38	0.66	6.61	–	0.16	2.11	7.27	0.87

**TABLE 2:** Elemental concentration in weight percentages obtained through scanning electron microscopy–energy‐dispersive X‐ray spectroscopy (SEM‐EDX) analysis

**Samples**	**O**	**Si**	**Pb**	**Al**	**Fe**	**C**	**K**	**Ca**	**Zn**	**Na**	**Mg**	**Cu**	**Co**	**Sn**	**Ni**	**Mo**	**Cr**	**Ti**	**As**
**GL 1**	28.93	16.38	14.28	6.16	4.78	0	1.5	3.52	1.24	0.95	1.24	1.57	—	3.68	0.66	—	0.87	0.66	0.47
**GL 2**	31.26	17.16	11.33	6.01	3.31	2.66	2.4	2.25	1.82	1.78	1.57	1.38	1.33	1.1	1.08	0.98	0.76	0.71	0.7
**GR 1**	29.57	11.06	9.77	4.26	2.91	4.44	0.82	11.82	0.88	—	1.05	0.55	0.84	7.86	0.42	0.56	0.65	0.64	0.41
**GR 2**	33.59	6.55	8.8	3.79	1.96	2.16	1.18	16.18	0.68	—	0.99	0.67	—	11.23	—	0.67	0.57	0.5	0.46

The presence of Pb in such high concentrations is because Pb is naturally found in copper ores. It is also possible that lead was applied to the slag to lower the smelting temperature. During the metal extraction process, when the copper ore is exposed to acidic slags, such as slags containing fayalite, silicate and other minerals, the lead is stripped from the metal,^21,^ ^22^ which also suggests the possibility of less amount of Pb present in the extracted copper metal. Also, the bulk of the sulphur gets removed when the ore was first roasted which was subjected to smelting for the removal of gangue material. When in the subsequent process of reducing the metal (copper matte), smelting, charcoal and flux were added during this stage, oxidation of sulphur took place to result in SO_2_ and Fe to FeO which was fluxed by silica (flux) to form the slag.^21^, ^22^ Also, iron and silica are mostly associated with copper ore which forms the slag.^12^

(3) FeO+SiO2↼⇁Fe−Si−Oslag

Surprisingly, EDX spectra did not show any presence of the Mn whereas XRF detected the presence of oxides of Mn. The oxides of Fe and Mn are indicators of reducing atmospheric conditions.^12^ The presence of quartz, fayalite and magnetite and the equilibrium between them indicated the redox conditions.^12^ Over content of silica might also be the reason for the presence of quartz. The molecular formula of fayalite also suggests the ideal composition of the slag in which Fe and Si concentrations are in a 2:1 ratio.^12^ Ca and Mg were also seen prominent in the XRD diagram of granulated slag because of which this type of slag has a dolomitic stoichiometry.^12^ The higher presence of Ca in the granulated slag may have stimulated the separation of Fe and Si from 2FeO.SiO_2_ for better slag reduction at high temperature, but a higher amount of Ca in the form of CaO may have also resulted in increasing the viscosity of the slag due to the higher presence of insoluble solid in the slag.^23^ The presence of magnesium and aluminium also indicates that their oxides might be the residues of magnetite present in the slag. Magnetite, apatite, quartz and chalcopyrite may be present as unreacted minerals.^24^

It has been reported that the earliest copper was produced from the smelting of oxides and carbonates and the smelting of sulfides was carried out in a much later period as sulfide smelting was much more complicated because it was very difficult to separate Cu from Fe and S. Although, some evidence reflect the use of extraction of copper from sulfides as Fe can help in complexing with silicates thus help in separating the siliceous gangue from the copper due to the formation of olivine or pyroxene.^12^ Therefore, the presence of olivine and pyroxene phases in the slags aligned with the elsewhere findings.^12^

### 3.5 ICP‐MS analysis

To further understand the elemental concentration and to identify the trace elements present in the slag, an ICP‐MS analysis was conducted. Measurement of the relative standard deviation of the data was helpful in ICP‐MS assessment and understanding the precision of the analytical procedure.^25^ Fourteen analytes and 5 liquid internal standards were obtained for the acquisition of the data (Table 3). All the analytes were selected based on the detected elements in SEM‐EDX analysis. To improve the sensitivity of the other analytes, all samples and calibration solutions were diluted so that the amount of copper is minimised in the solution.^26^ The concentration of all analytes was higher in both glassy slag samples than in granulated slag samples. However, the concentration of calcium was much higher in granulated slags. This indicated the difference in nature of both types of slag samples and supported the chemical character and phase composition obtained through SEM‐EDX and XRD respectively. This further corroborated the dolomitic character of the granulated slags. The major concentration found of Mg, Al, Ca and Fe occurred at high concentrations. The element of minor concentration was Ti, Pb, Cr, Co, Cu, Zn, As, Mo, Sn and Bi. The opposite result was observed in the case of Pb in SEM‐EDX and XRF analyses, where Pb was identified as one of the major elements. Fe concentrations were heavy in glassy slags. Pb concentrations were found to be lower using ICP‐MS. This may be due to the ICP‐MS method's inefficiency in detecting Pb as lead being a high mass analyte and also requires a high amount of sample (2–5 g) for digestion in concentrated nitric acid or aqua regia.

**TABLE 3:** Elemental concentration in part per billion of 14 analytes measured with inductively coupled plasma–mass spectrometry (ICP‐MS)

**Analytes**	**Conc (in ppb) GL 1**	**GL2**	**GR 1**	**GR 2**
24 Mg	26,669.462	26,524.027	34,818.575	27,205.431
27 Al	155,404.695	147,542.429	60,163.327	80,677.055
43 Ca	15,896.268	15,463.634	116,718.112	108,382.71
47 Ti	2763.077	2847.589	3777.581	4117.118
52 Cr	910.257	403.149	126.093	208.671
56 Fe	146,448.647	178,956.317	60,817.614	87,311.547
59 Co	88.353	91.95	29.418	29.609
63 Cu	3644.125	12,270.118	57.716	60.554
66 Zn	489.307	702.291	196.724	191.453
75 As	16.089	21.144	25.32	45.409
95 Mo	13.465	3.982	3.817	3.897
118 Sn	10.95	18.159	4.61	5.153
208 Pb	752.949	870.393	242.899	255.88
209 Bi	0.331	0.312	1.022	1.183

GL = Glassy slag; GR = Granulated slag.

XRF and ICP‐MS both showed the iron levels to be very high. A high concentration of iron means that the smelted slag has been properly smelted, while a low concentration of iron indicates that the smelted slag has been poorly reduced due to the smelting furnace's low temperature. This also implies that the smelting furnace was capable of reaching a high temperature and providing a strongly reducing environment.^27^ The primitive smelting technology included the crushing of slags by hammering with stone and smelting in a poorly reduced condition. But the high presence of iron indicates that the reducing condition was much better.^27^ Furthermore, given the heterogeneity of the slags, it indicated that the smelting temperature might not have reached the point where the slag is completely fluid.^27^ Due to the heterogeneity in the chemical composition of the slags, XRF, SEM‐EDX and ICP‐MS produced varying percentages of elemental composition.

### 3.6 Smelting conditions

According to archaeological evidence, copper pyrites were heated in closed crucibles and before they were heated in a smaller blast furnace with hand bellows to produce the blast, they were roasted with charcoal mixed with flux.^1^ The bellows would help in increasing the level of oxygen for achieving the higher temperature and which also allowed controlling the increase the temperature of oxygen to avoid providing the reducing condition for the reduction of iron minerals in the furnace.^28^ When the copper is obtained from the sulfide ores such as chalcopyrite, it yields low percentages of copper that also requires undergoing separation by the froth flotation process before the refining process. During this process, most of the sulphur present in the ore gets converted into sulphur dioxide gas. The top portion of the slag that is in contact with the atmosphere solidifies quickly into mineral glass form and the underneath layers cool slowly and forms crystalline structures. Both upper and inner layers of slags remain similar in chemical composition.^1^

It's fascinating to learn how the people of the Harappan civilization dealt with slags at the time. As a result of improper slag disposal, slags can mix with the soil and be carried by the wind, posing a significant health risk to the local public, as Cu contamination may cause carcinogenic risk.^29^ Geochemical characteristic of slag is also important to understand whether the metal content is high in the slag residue and can be reused.^29^ It gives us a possibility that the ores were collected from the nearby deposits but that relationship cannot be certainly ascertained. Similar lumps were also found in other parts of the country where copper objects were found, the evidence of which indicated that the smelting was done near the site of the recovered slag.

## 4 CONCLUSION

Based on the analytical results obtained, the presence of dominant mineral phase fayalite as per the XRD measurement clearly indicated the reducing conditions of the smelting process. The reducing atmosphere was further corroborated by the presence of iron in higher concentrations. The presence of calcium in higher concentrations was shown by SEM‐EDX, XRF and ICP‐MS, the granulated slag showed a dolomitic character. The very less weight percentage of copper obtained through EDX revealed the high ancient smelting technology. Since the extraction method was highly efficient, there was a very low percentage of copper in the slag. Silica seems to have been used as a fluxing material, and the slag formed a high degree of glassy phase as a result of the flux, which could aid in efficiently separating the copper from the slag with a higher yield. The slag also showed the presence of some precious metals such as cobalt, nickel, titanium, etc. There was also no possibility of iron recovery because the slag was cooled in a slow cooling process. There was no evidence of a slag recycling operation. In this research, we showed the absence of sulphur which is unique to the study of copper slag.

## CONFLICT OF INTEREST

The authors declare no conflict of interest.

## Data Availability

Data generated during the research can be made available on request.
